# Combinatorial therapeutic targeting of BMP2 and MEK-ERK pathways in NF1-associated malignant peripheral nerve sheath tumors

**DOI:** 10.18632/oncotarget.11036

**Published:** 2016-08-03

**Authors:** Sidra Ahsan, Yubin Ge, Michael A. Tainsky

**Affiliations:** ^1^ Cancer Biology Graduate Program, Wayne State University School of Medicine, Detroit, MI 48201, USA; ^2^ Department of Oncology, Wayne State University School of Medicine, Detroit, MI 48201, USA; ^3^ Molecular Therapeutics Program, Karmanos Cancer Institute, Wayne State University School of Medicine, Detroit, MI 48201, USA; ^4^ Center for Molecular Medicine and Genetics, Wayne State University School of Medicine, Detroit, MI 48201, USA

**Keywords:** neurofibromatosis type 1, malignant peripheral nerve sheath tumors, BMP2, combinatorial targeted therapy, cell signaling

## Abstract

The clinical management of malignant peripheral nerve sheath tumors (MPNSTs) is challenging not only due to its aggressive and invasive nature, but also limited therapeutic options. Using gene expression profiling, our lab identified BMP2-SMAD1/5/8 pathway as a potential therapeutic target for treating MPNSTs. In this study, we explored the therapeutic impact of targeting BMP2-SMAD1/5/8 pathway in conjunction with RAS-MEK-ERK signaling, which is constitutively activated in MPNSTs. Our results indicated that single agent treatment with LDN-193189, a BMP2 Type I receptor inhibitor, did not affect the growth and survival of MPNST cells at biochemically relevant inhibitory concentrations. However, addition of a MEK1/2 inhibitor, selumetinib, to LDN-193189-treated cells resulted in significant inhibition of cell growth and induction of cell death. LDN-193189 at biochemically effective concentrations significantly inhibited motility and invasiveness of MPNST cells, and these effects were enhanced by the addition of selumetinib. Overall, our results advocate for a combinatorial therapeutic approach for MPNSTs that not only targets the growth and survival via inhibition of MEK1/2, but also its malignant spread by suppressing the activation of BMP2-SMAD1/5/8 pathway. Importantly, these studies were conducted in low-passage patient-derived MPNST cells, allowing for an investigation of the effects of the proposed drug treatments in a biologically-relevant context.

## INTRODUCTION

Neurofibromatosis Type I (NF1) syndrome is one of the most common heritable genetic conditions of the nervous system with a birth incidence of 1 in 3000 individuals [1]. The NF1 syndrome is characterized by mutations in the neurofibromin I (*Nf1*) gene. Heterozygous inheritance of a defective *Nf1* gene leads to a wide variety of clinical pathologies including café-au-lait macules, axillary freckling, Lisch nodules, cognitive disorders, bone deformities, and neurofibromas [2]. NF1 patients are also susceptible to various forms of cancers, including glioma of the optic pathway, gastrointestinal stromal tumors, rhabdomyosarcomas, leukemia, breast cancers, etc. [3]; development of which requires a complete loss of *Nf1* gene function [4]. Although all these cancers present with poor prognosis in NF1 patients, malignant peripheral nerve sheath tumor (MPNST) is the most aggressive cancer seen in NF1 patients with a five-year survival rate of 21% [5]. MPNSTs originate from Schwann cells associated with the peripheral nerves, and account for 5-10% of all soft tissue sarcomas [6]. MPNSTs may occur sporadically or in association with the NF1 syndrome. Up to half of MPNST cases are diagnosed in people with the NF1 disease [7], and 41% of the remaining sporadic MPNST cases present with sporadic mutations in the *Nf1* gene [8], highlighting the role of *Nf1*-mediated deregulation in the biogenesis of MPNSTs. *Nf1 is* a tumor suppressor gene due to its well-characterized Ras GTPase activating protein related domain (RAS-GRD), which negatively regulates RAS activity by accelerating the hydrolysis of the activated GTP-bound RAS [9]. Thereby, neurofibromin deficiency results in activation of the wild-type Ras proto-oncogenes that play a central role in development and maintenance of NF1 syndrome-related tumors. The activation of downstream effectors of Ras signaling such as MEK1/2 occurs in 91% of MPNST patient tissue samples, as compared to 21% of benign neurofibromas [10], and contributes to the proliferation and survival of MPNST cell lines [11].

Although surgery is the primary treatment option for MPNSTs, its success is limited by tumor infiltration resulting in a high relapse rate. Due to the size and location of MPNSTs, surgery is performed with wide margins, but often unfortunately leaving behind cancer cells needing additional chemotherapy [12]. Currently, there are no chemotherapeutic regimens that effectively treat MPNSTs. Doxorubicin and ifosfamide have traditionally been used as the chemotherapy regimen for MPNSTs; however, a ten-year institutional review showed no correlation between chemotherapy and patient survival [13]. Due to the failure of conventional chemotherapy, there has been a trend towards therapies that target the altered cellular signaling in MPNSTs specifically the Ras-associated pathways. However, results from the clinical evaluation of inhibitors of the Ras pathway have been disappointing. Tipifarnib, a farnesyl transferase inhibitor (FTI) that blocks the prenylation step in activation of the Ras protein and its association with the cellular membrane, failed in Phase II clinical trials in young NF1 patients with plexiform neurofibromas, as geranylgeranyltransferase compensated for the inhibition of prenylation of N-RAS and K-RAS by FTIs [14, 15]. BRAF inhibitors, such as sorafenib exhibited significant toxicity in NF1 patients in clinical trials [16], whereas mTOR inhibitor sirolimus did not affect tumor burden, although it prolonged time to disease progression by four months in plexiform neurofibroma patients [17]. Conversely, selumetinib, an ATP-independent inhibitor of MEK1/2, has shown promising results in clinical trials for young adults with inoperable plexiform neurofibromas in association with the NF1 syndrome [NCT02407405] (48). Moreover, it was recently approved by the U.S. Food and Drug Administration (FDA) for the treatment of uveal melanomas, the majority of which harbor mutations that behave similarly to *BRAF* mutations and result in constitutive activation of the MAPK pathway [18, 19]. Selumetinib has proven patient tolerance in clinical trials of various cancers, especially those dependent on increased MEK-ERK signaling, however, its effect as a single drug seems to be limited [20]. Due to the inherent genomic complexity of NF1 syndrome-associated MPNSTs, therapy with a single targeted agent may not be efficacious, and therefore a rationally designed combinatorial approach that targets multiple disease-related survival pathways is the obvious option for a more effective treatment and management of these tumors. Additionally, the functions of other domains of neurofibromin are not clear. Interestingly, various single nonsense and missense mutations in the *Nf1* gene outside the GRD sequence can lead to NF1 disease manifestation in patients [21]. Another study reported that induced expression of *Nf1* RAS-GRD does not rescue the lethality associated with *Nf1*^(−/−)^ mouse models [22], suggesting that NF1 regulates vital mechanisms of development and tumorigenesis, independently of RAS-GRD.

Currently, therapeutic options for MPNSTs or unresectable plexiform neurofibromas do not address the metastases of MPNSTs, which occur in over 40% of MPNST patients [23]. Using gene expression profiling, our lab has identified the BMP2-SMAD1/5/8 signaling pathway as an NF1-dependent regulator of motility and invasiveness in MPNST cell lines, independent of the RAS-MEK1/2 signaling pathway [24]. Based on amino acid sequence homology, BMP2 belongs to the transforming growth factor beta (TGF-β) family [25]. BMP2 is a secreted protein that signals via hetero-oligomeric complexes of serine/threonine kinase receptors, type I and type II receptors. Specifically, BMP2 signals through three type I receptors: BMPR-IA or ALK-3, BMPR-IB or ALK-6, and ActR-IA or ALK-2 [26]. Upon ligand binding, type I and type II receptors form a hetero-tetrameric receptor complex [27] that initiates a signaling cascade involving SMAD family of proteins. SMAD 1, 5 and 8 are phosphorylated by the BMP receptor complex and then associate with SMAD4 [28]. The SMAD1/5/8-SMAD4 complex translocates to the nucleus and initiates gene transcription in a tissue and developmental stage-specific fashion [27, 29]. The role of BMP2 in progression to MPNSTs is evident by changes in its expression levels at various stages of neurofibroma development in clinical specimens. Our data mining of publicly available transcriptional profiling of NF1 syndrome-related clinical specimens demonstrated that *Bmp2* expression was significantly higher in plexiform neurofibromas and MPNST patient samples, as compared to the benign forms of neurofibromas [24]. Approximately 56-57% of all NF1 patients develop plexiform neurofibromas which are extensive, larger neurofibromas that can occur anywhere within the body [21, 30] and can transform into the aggressive MPNSTs. Therefore, BMP2 may represent a promising therapeutic target for treating MPNSTs.

In this study, we present *in vitro* data for targeting of the BMP2-SMAD1/5/8 pathway in combination with inhibition of the MEK-ERK pathway as a treatment strategy for MPNST patients. The rationale behind this approach is to target the viability, growth, and proliferation of MPNSTs by inhibition of the MEK1/2-ERK1/2 pathway, and inhibit motility and invasiveness of MPNSTs via inhibition of the BMP2-SMAD1/5/8 signaling. Our results suggest that the combinatorial targeting of these molecular alterations results in increased cell death, decreased invasion and migration of low passage MPNST cells, an overall reduction of the malignant potential of MPNST cells.

## RESULTS

### BMP2 is overexpressed in low and high passage *Nf1*-null MPNST cell lines

**Figure 1:**
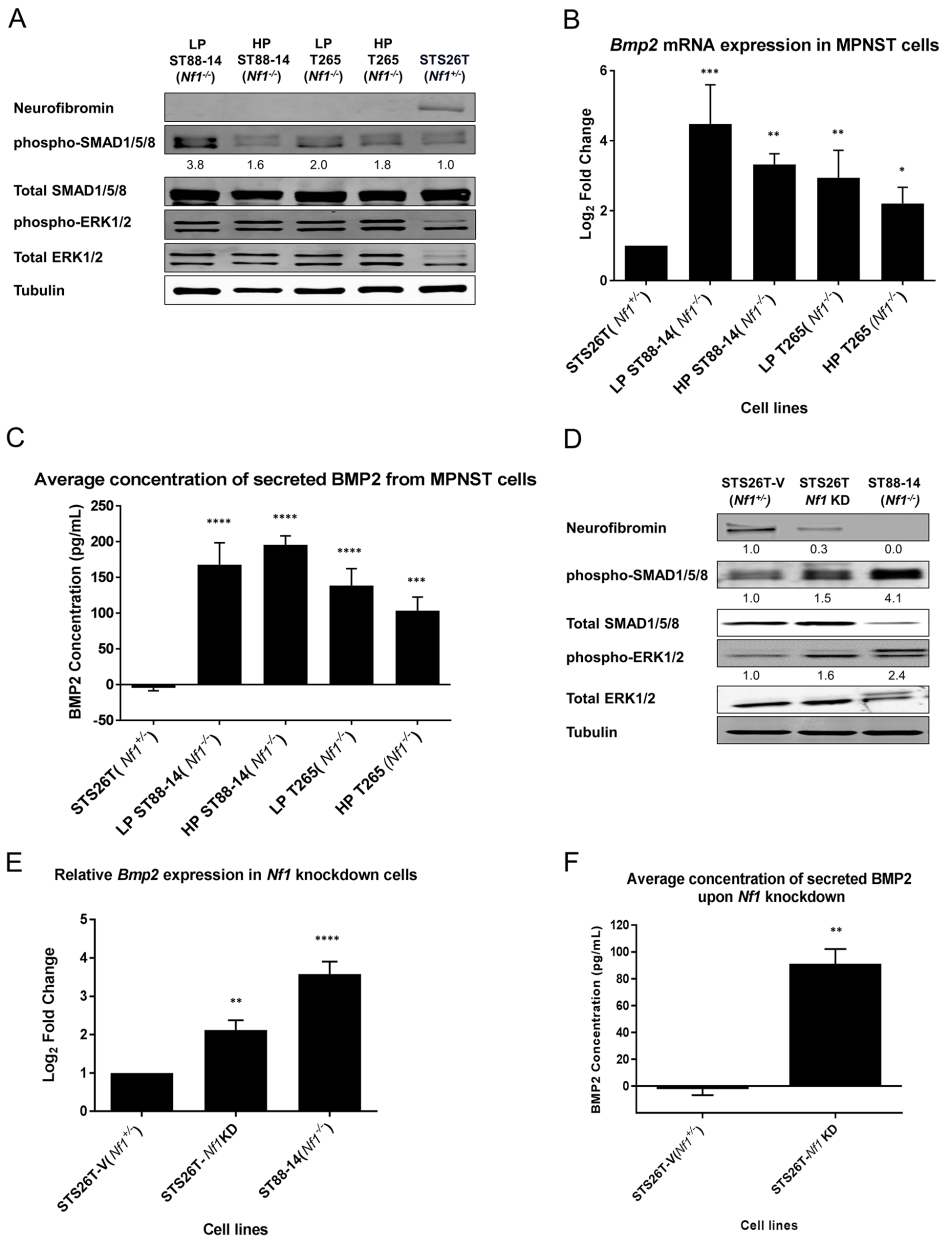
BMP2 is overexpressed in NF1-null MPNST cell lines **A.** Representative western blot (n=3), of LP and HP (*Nf1^−/−^*) MPNST cell lines confirmed their *Nf1*-null status and shows an active BMP2-SMAD1/5/8 pathway and MAPK pathway. Quantification of the western blot reveals that there is at least a 1.6-fold increase in phospho-SMAD1/5/8 activity in all the *Nf1^(−/−)^* cell lines as compared to the positive control STS26T (*Nf1^+/−^*) cells, with the highest levels reported in the low passage *Nf1*-null cells. The quantification values were calculated by normalizing densitometry measurements of phospho-SMAD1/5/8 to total SMAD1/5/8 and then compared to STS26T (*Nf1^+/−^*) cells (set as 1). **B.** In *Nf1*-null MPNST cells, the expression of *Bmp2* is significantly higher irrespective of passage numbers as compared to the STS26T (*Nf1^+/−^*) cells. RNA was extracted between passages 8-14 from LP ST88-14 (*Nf1^−/−^*), passages 155-170 for HP ST88-14 (*Nf1^−/−^*), passages 10-16 for LP T265 (*Nf1^−/−^*) cells, and passages 208-230 for HP T265 (*Nf1^−/−^*) cells. **C.** Analyses of BMP2 secretion by ELISA using conditioned media from MPNST cell lines shows that BMP2 is secreted in both low and high passage *Nf1*-null MPNST cells. **D.** Activation of BMP2-SMAD1/5/8 and MEK1/2-ERK1/2 pathway is dependent on the presence of neurofibromin I, as both pathways are activated via phosphorylation upon knockdown of *Nf1*. Based on densitometry, a ~70% knockdown of NF1 results in a 55% increase in phospho-SMAD1/5/8 levels, and a 60% increase in the phospho-ERK1/2 levels. The relative levels of NF1, phospho-SMAD1/5/8, and phospho-ERK1/2 were calculated by normalizing their densitometry measurements to the corresponding tubulin, total SMAD1/5/8, and total ERK1/2, respectively. **E.** Results from RT-PCR show that *Bmp2* expression increased two-fold upon *Nf1* knockdown as compared to the STS26T-V (*Nf1^+/−^*) cells. All cell lines used in this experiment are high passage cells. The *Nf1*-knockdown cells were generated at passage 160 for the STS26T-V (*Nf1^+/−^*) cells, and were maintained in culture for subsequent 8-10 weeks. Steady-state *Bmp2* mRNA levels of ST88-14 (*Nf1^−/−^*) cells were determined between passages 175-190. **F.** Secreted levels of BMP2 are also dependent on the status of NF1, where knockdown of *Nf1* results in increased levels of secreted BMP2. All cell lines used in this experiment are high passage cells. The *Nf1*-knockdown cells were generated at passage 160 from the STS26T cells, and were maintained in culture for subsequent 8-10 weeks. Paired t-test or one-way ANOVA followed by Tukey's test for multiple comparisons used for determining statistical significance (n=3, *P<0.05, **P<0.01, ***P<0.001, ****P<0.0001 as compared with either STS26T (*Nf1^+/−^*) or STS26T-V (*Nf1^+/−^*), as specified).

Our lab has previously reported a significant increase in *Bmp2* expression levels in long-term cultures of *Nf1^(−/−)^* MPNST cell lines, independent of the RAS-MEK1/2-ERK1/2 regulation [24]. Cells in culture are constantly subjected to environmental and manipulative stresses, which may introduce genotypic and phenotypic variations by selection of a dominant clone. These alterations in the cellular signaling pathways introduced by prolonged cell culturing are well-documented in literature [31, 32]. To circumvent the inherent bias of the cell culture system, low passage (LP) patient-derived MPNST cells, LP ST88-14 (*Nf1^−/−^*) and LP T265 (*Nf1^−/−^*) cell lines were used in this study to validate the role of BMP2 in MPNSTs. Western blotting analysis of low and high passage (HP) MPNST (*Nf1^−/−^*) cells confirmed the status of NF1 protein in these cell lines, and revealed an active BMP2-SMAD1/5/8 pathway via detection of phospho-SMAD1/5/8, as compared to the positive control STS26T (*Nf1^+/−^*) cells that express the neurofibromin I protein (Figure 1A). Although all *Nf1*-null cell lines had higher levels of phospho-SMAD1/5/8 than the STS26T (*Nf1^+/−^*) cells, these levels were more pronounced in the physiologically relevant low passage cells (Figure 1A). As one would expect, increased phosphorylation of ERK1/2 in all of the tested cell lines was also detected, indicative of an active MEK-ERK pathway.

To determine the effects of cell passage on the expression levels of BMP2, we first measured the transcript levels for *Bmp2* by real-time RT-PCR. Interestingly, both LP and HP *Nf1*-null cells showed significantly higher *Bmp2* transcript levels as compared to the STS26T (*Nf1^+/−^*) cells (Figure 1B). Although the transcript levels for *Bmp2* in HP cells tended to be lower than in the LP cells, the differences were not statistically significant. Further, the cell passage did not have an obvious impact on the levels of secreted BMP2 protein in the *Nf1*-null cell lines, which were significantly higher than in the STS26T (*Nf1*^+/−^) cells (Figure 1C).

The change in expression of BMP2 was dependent on the status of NF1 protein, in which down-regulation of NF1 protein resulted in activation of SMAD1/5/8 (Figure 1D) and a two-fold increase in *Bmp2* transcript levels (Figure 1E). Analysis of conditioned media from *Nf1*-knockdown and *Nf1^(+/−)^* cell lines indicated that the secretion of BMP2 was also dependent on the status of NF1 protein (Figure 1F). The TGF-β - SMAD1/5/8 pathway may be activated by other members of the BMP sub-family, such as BMP4, BMP7, BMP9, etc. Based on pathway analysis of the gene expression profiling data, we identified BMP2 as the principal mediator of TGF-β pathway in NF1-related MPNSTs, upon knockdown of NF1. Other BMP family members did not exhibit significant changes in gene expression levels upon *Nf1* knockdown, whereas BMP4, the closest homolog of BMP2, was down-regulated (data not shown). These results validate our previous findings that upon down-regulation of NF1 protein, BMP2 is up-regulated in MPNST cells and results in activation of SMAD1/5/8 pathway. Additionally, these data corroborate gene expression profiling of MPNST patient tissue samples by an independent group [33] in which we found that BMP2 is up-regulated in MPNSTs as compared to other benign forms of neurofibromas that are typically heterozygous for *Nf1* [24].

### Combinatorial inhibition of BMP2 and MEK-ERK signaling results in increased cell death of MPNST cells

**Figure 2:**
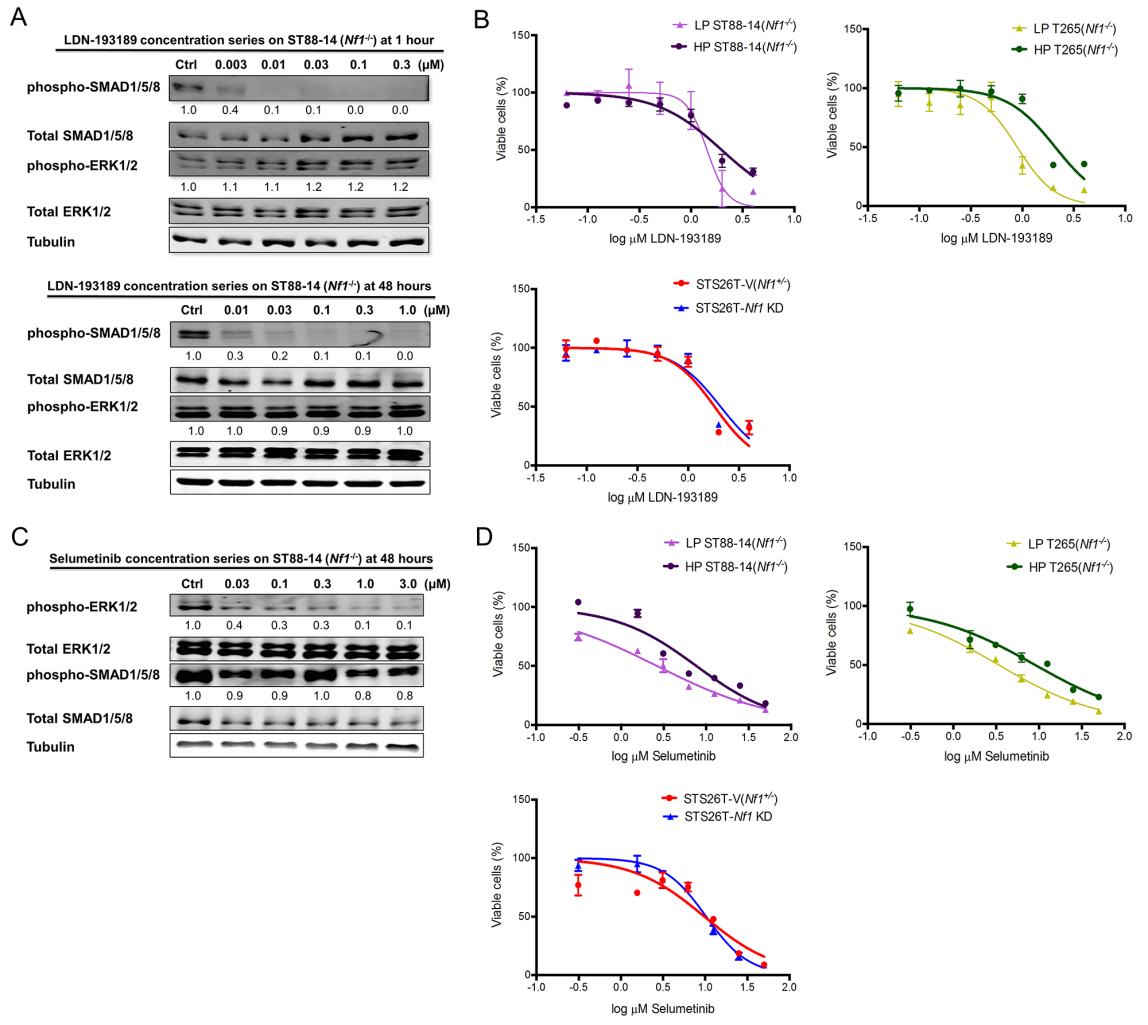
Single agent treatment with LDN-193189 or selumetinib affects cellular viability at concentrations higher than the biochemically effective dose **A.** Representative western blot (n=3) of titration of LDN-193189 in MPNST cells to determine optimal dose of inhibition. LDN-193189 potently inhibits phospho-SMAD signaling at 0.003 μM with nearly complete inhibition at 0.01 μM, without any effects on the ERK signaling pathway in the ST88-14 (*Nf1^−/−^* cells) at the 1-hour time point. LDN-193189 continues to inhibit phospho-SMAD1/5/8 at the low concentration of 0.01 μM in 48 hours, however, it requires between 0.03 and 0.1 μM for complete inhibition of the target in 48 hours. None of the tested concentrations of LDN-193189 up to 1.0 μM had any effects on the MEK1/2-ERK1/2 signaling. The relative levels for phospho-SMAD1/5/8 and phospho-ERK1/2 were calculated by normalizing their densitometry to the corresponding total SMAD1/5/8 and total ERK1/2, respectively. Similar concentration responses were obtained for all tested MPNST cell lines. Cells were treated with the indicated concentrations for the specified time points and whole cell lysate was fractionated on SDS-PAGE followed by immunoblotting for indicated proteins, n=3. **B.** The IC_50_ for single agent treatments were plotted on a semi-logarithmic scale in which x-axis (log_10_ scale) indicates drug concentrations and y-axis represents % of viable cells as measured by 48-hour MTT assays. In all the tested cell lines, the IC_50_ for LDN-193189 was between 1.0-2.0 μM. Data were analyzed by non-linear regression analysis to generate sigmoidal dose response curves and each point represents mean value from three independent experiments ± SD. **C.** Representative western blot (n=3) showing that selumetinib inhibits phospho-ERK1/2 in a concentration-dependent manner. Concentrations between 0.03 μM to 0.1 μM lead to a significant decrease in activation of ERK1/2 in 48 hours. The relative levels of phospho-ERK1/2 and phospho-SMAD1/5/8 were calculated by normalizing their densitometry to the corresponding total ERK1/2 and total SMAD1/5/8, respectively. **D.** The effects of single treatment by selumetinib on the percent of viable MPNST cell lines. The IC_50_s for selumetinib treatments were plotted on a semi-logarithmic scale in which x-axis (log_10_ scale) indicates drug concentration and y-axis represents % of viable cells as measured by 48-hour MTT assays. Data were analyzed by non-linear regression analysis to generate sigmoidal dose response curves and each point represents mean value from three independent experiments ± SD.

Currently, there are no FDA approved BMP2 receptor inhibitors. LDN-193189 is the most specific BMP2 activated Type I receptor inhibitor. It has at least a 100-fold selectivity for type I receptors (ALK2, ALK3 and ALK6) over other receptors of the TGF-β pathway [34]. Upon binding to the Type I receptors, LDN-193189 suppresses the hetero-oligomerization of the Type I and Type II serine/threonine kinase receptors leading to the inhibition of phosphorylation of the SMAD1/5/8 complex [35]. For BMP2, receptor oligomerization is the critical step initiating SMAD1/5/8 signaling [36]. To investigate the effect of targeting BMP2-SMAD1/5/8 signaling on the growth and survival of MPNST cells, we evaluated the inhibition efficacy of LDN-193189 at different concentrations. We found that treatment of ST88-14 (*Nf1^−/−^*) cells with 0.01 μM LDN-193189 for 1 hour resulted in nearly complete inhibition of phospho-SMAD1/5/8, and these effects were sustainable for 48 hours (Figure 2A). Next, we determined the IC_50_ of LDN-193189 in various MPNST cell lines. We found that LDN-193189 inhibited the growth of MPNST cells at concentrations approximately 100-fold higher than the biochemically effective concentration required for inhibition of the BMP2-SMAD1/5/8 pathway. While within 48 hours, between 0.01 μM and 0.03 μM of LDN-193189 were sufficient for inhibition of BMP2 signaling, LDN-193189 IC_50_ ranged from ~1.0-2.0 μM as a single agent in various MPNST cell lines (Figure 2B). These results suggest that the effects of higher concentrations of LDN-193189 on the growth of MPNST cell lines are potentially off-target effects. To enhance the effect of biochemically relevant concentrations of LDN-193189 on cell growth and survival, we chose to target the MEK-ERK pathway in combination with BMP2 signaling. MEK1/2 are critical therapeutic targets in treatment of MPNSTs based on their extensively documented role in progression and maintenance of MPNSTs [37]. Moreover, preliminary data from the Phase I study of selumetinib on plexiform neurofibromas in NF1 patients suggested that treatment with selumetinib resulted in a median decrease of 24% in the volume of plexiform neurofibromas with reversible toxicities in all patients with >1 MRI restaging (48). Treatment of ST88-14 (*Nf1*^−/−^) with selumetinib resulted in concentration-dependent down-regulation of phospho-ERK1/2, but had no obvious effects on the levels of phospho-SMAD1/5 (Figure 2C). Next, we determined selumetinib IC_50_s of MPNST cell lines. Low passage ST88-14 (*Nf1^−/−^*) and T265 (*Nf1^−/−^*) cells were more sensitive to selumetinib treatment with IC_50_s ranging from ~3 to 4 μM as compared to ~8 to 9 μM for their high passage counterparts and 10 μM for the STS26T cell lines (Figure 2D). Similar to LDN-193189, selumetinib required a higher concentration to affect cell growth and survival than the biochemical concentration required to inhibit its target in 48 hours.

**Figure 3:**
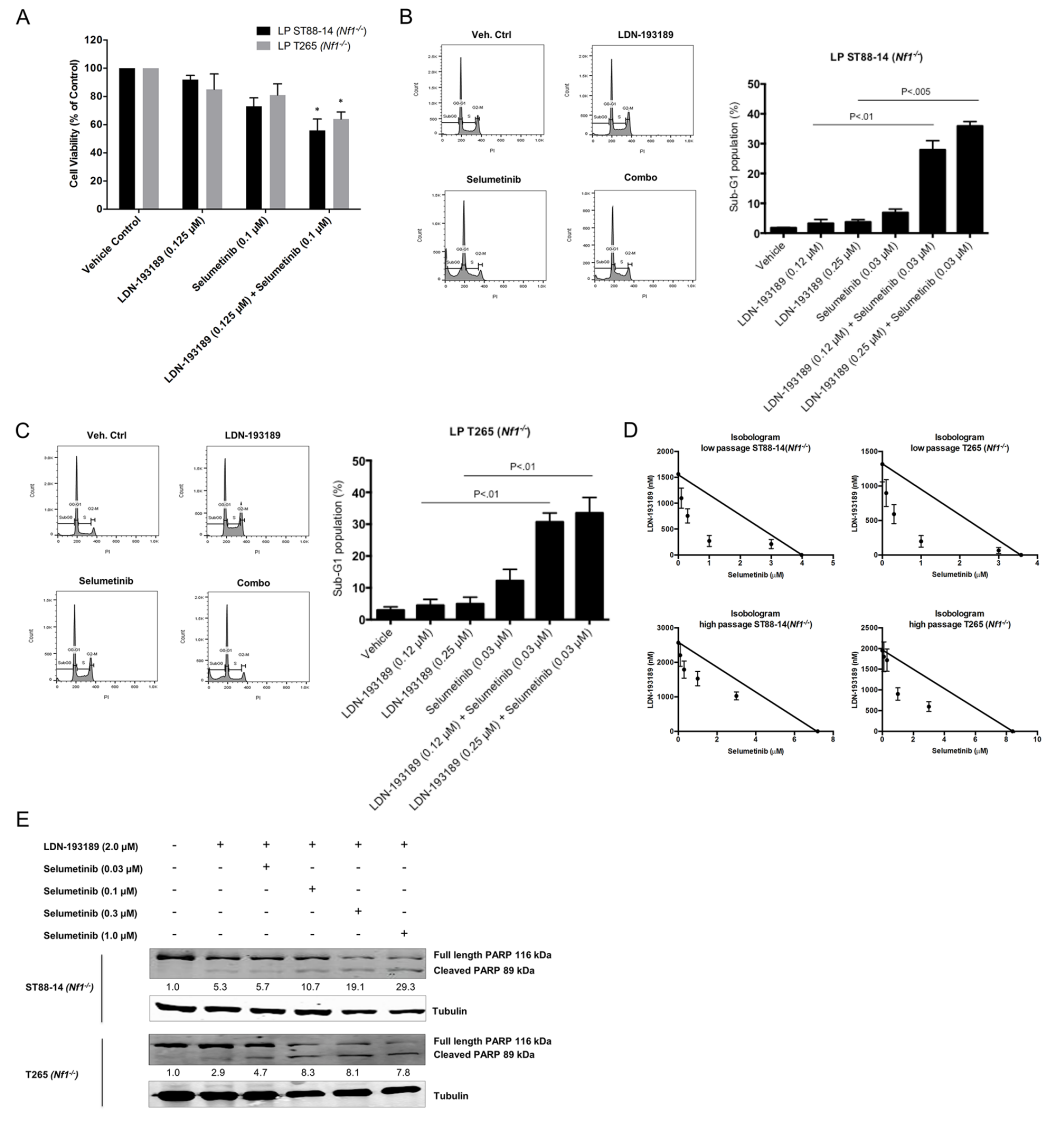
Combinatorial treatment with LDN-193189 and selumetinib results in increased cell death and decreased proliferation of MPNST cells **A.** Bar graph of the percentage of viable low passage MPNST cells via MTT assay. Cells were treated with the indicated drug concentrations for 48 hours. Single agent treatment with LDN-193189 does not affect cell growth at concentrations below its IC_50_, however addition of selumetinib significantly decreases the % of viable MPNST cells (n=3, *P<0.05, paired t-test compared to cells treated with LDN-193189 only). **B.** and **C.** Representative histograms from cell cycle analyses of low passage NF1-null MPNST cell lines treated with either single agent or combination agents for 48 hours. Bar graph from cell cycle analyses data of MPNST cells treated with biochemically relevant concentrations of LDN-193189 and selumetinib. Percentage of cells in sub-G1 phase increase upon combinatorial treatment with LDN-193189 and selumetinib as compared to single treatment with either of the agents (n=3, paired t-test combination treatment compared to cells treated with LDN-193189 only). **D.** Standard isobologram analyses of the anti-tumor interactions between LDN-193189 and selumetinib in various MPNST cell lines. All drug combinations exhibit a synergistic effect in low and high passage NF1-null MPNST cells with an increased synergistic interaction in the low passage cells. Each axis represents the indicated concentrations of that drug. Data points represent the average value from three independent experiments ± SD. **E.** Results from PARP cleavage assay upon 48-hour treatment with candidate drugs indicate a dose-dependent induction of apoptosis upon combination treatment as compared to treatment with LDN-193189 alone. For treatment with LDN-193189 alone, cells were treated with 2.0 μM of LDN-193189 based on the drug's IC_50_ as determined by the MTT assay. For combination treatment, cells were treated with 2.0 μM of LDN-193189 in combination with biochemically effective increasing concentrations of selumetinib. The relative levels of cleaved PARP were calculated by normalizing their densitometry measurements to tubulin, and then compared to vehicle-treated control (set as 1).

In order to avoid off-target effects, we tested the impact of combination treatment with LDN-193189 and selumetinib at biochemically relevant concentrations on the growth and survival of MPNST cells. Addition of selumetinib to MPNST cells treated with LDN-193189 resulted in a statistically significant decrease in viable cells (Figure 3A). Furthermore, cell cycle analyses of low passage cells treated with either agent alone or in combination revealed that combination of the two agents resulted in cooperative induction of cell death, reflected by the sub-G1 cell populations (Figure 3B & 3C). Interestingly, single agent treatment with either of the agents required higher than the biochemically effective concentration to affect cell growth, but the combined inhibition of BMP2 and MEK1/2 at biochemically effective concentrations led to growth inhibition and induction of cell death. Moreover, evaluation of the nature of the drug interaction between LDN-193189 and selumetinib through isobologram analyses (Figure 3D) and calculation of combination index (CI) values (Table 1) demonstrated that the dual treatment with LDN-193189 and selumetinib resulted in a strong synergic effect (CI<1.0) on cell growth of both low passage MPNST cell lines. Both candidate agents synergized to inhibit cell growth in the long-term cultures of *Nf1*-null MPNST cells as well as the sporadic MPNST cells (Table 1). To elucidate the mechanism by which the combination of LDN-193189 and selumetinib induces cell death, ST88-14 (*Nf1^−/−^*) and T265 (*Nf1^−/−^*) cells were treated with the drug combination, and levels of PARP cleavage were measured using western blots. The dose-dependent increase in levels of cleaved PARP and the corresponding decrease in levels of full-length PARP suggest induction of apoptosis in response to combination treatment (Figure 3E).

**Table 1:** Combination Index (CI) values represent synergy of selumetinib and LDN-193189 in MPNST cell lines

Combination drug treatments	MPNST cell lines					
	STS26T-V *(Nf1^+/−^)*	STS26T-*Nf1* KD	LP ST88-14 *(Nf1^−/−^)*	HP ST88-14 *(Nf1^−/−^)*	LP T265 *(Nf1^−/−^)*	HP T265 *(Nf1^−/−^)*
LDN-193189 +0.1 μM Selumetinib	0.73	0.65	0.58	0.85	0.62	0.99
LDN-193189 +0.3 μM Selumetinib	0.64	0.61	0.39	0.86	0.48	0.99
LDN-193189 +1.0 μM Selumetinib	0.55	0.74	0.32	0.91	0.37	0.71
LDN-193189 +3.0 μM Selumetinib	0.68	0.67	0.92	0.84	0.78	0.65

CalcuSyn software was used to calculate the CI, according to the median-effect method of Chou-Talay. CI values less than 1, 1, and greater than 1, represent synergism, additivity, and antagonism, respectively. The combinatorial treatment has a synergistic effect on cell growth in the STS26T-V (*Nf1^+/−^*) and STS26T-*Nf1* KD cell lines. Both LP ST88-14 (*Nf1^−/−^*) and T265 (*Nf1^−/−^*) cells exhibit a strong synergistic interaction at lower doses of selumetinib combined with LDN-193189. HP ST88-14 (*Nf1^−/−^*), and HP T265 (*Nf1^−/−^*) trend towards additivity at lower concentrations of selumetinib, however, increasing the dose of selumetinib increases the synergy in these cell lines. The CI values shown are averages of CI values of three independent experiments per cell line and treatment conditions.

### Selumetinib cooperates with LDN-193189 to decrease cellular invasion and migration of MPNSTs

**Figure 4:**
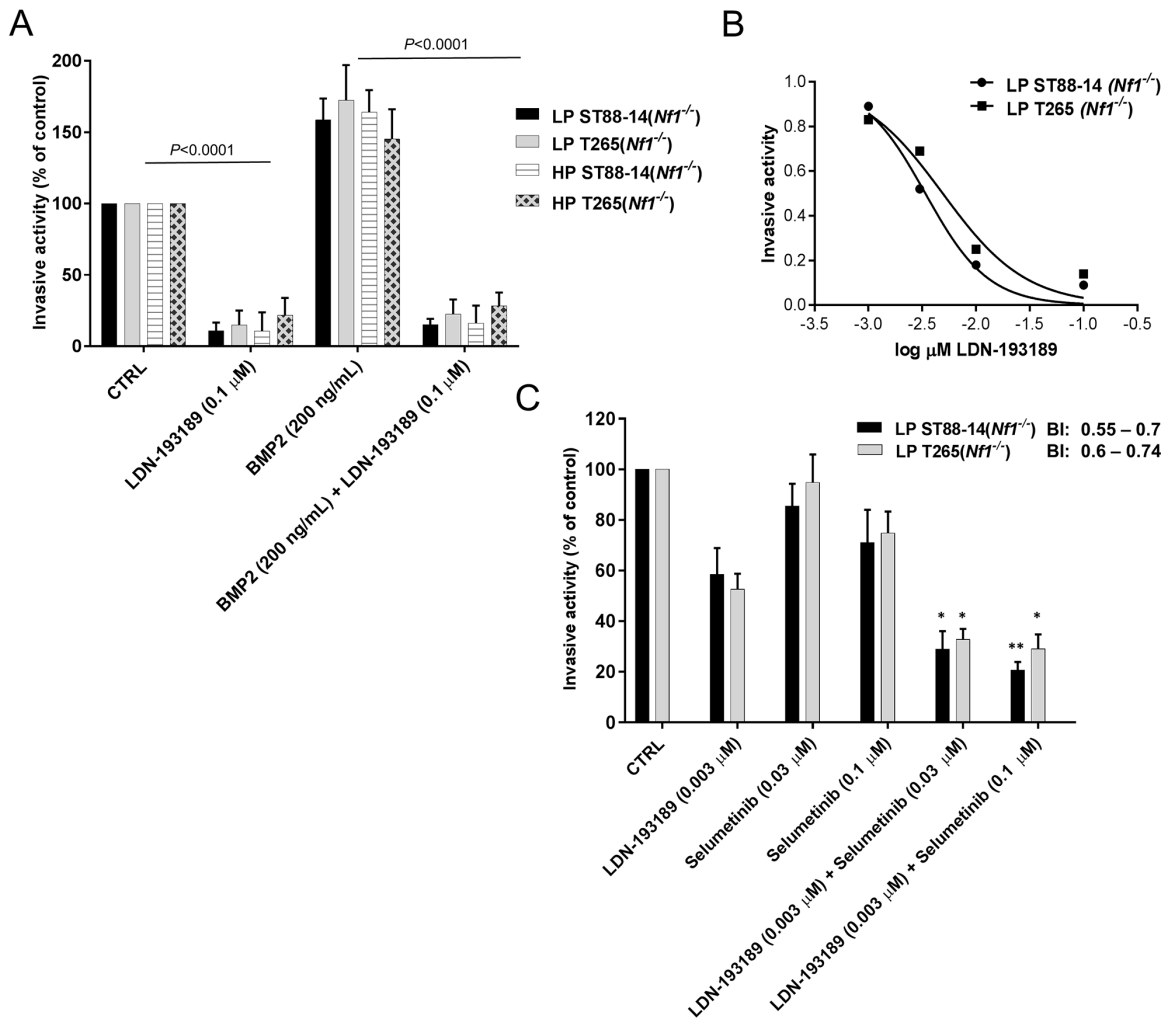
Combinatorial treatment with LDN-193189 and selumetinib synergizes to reduce cellular invasion in MPNST cells **A.** Graphical representation of the quantified fluorescence of the cells that invaded through the extracellular matrix (ECM), normalized to invasive activity without any chemoattractant and the vehicle control. LDN-193189 inhibits cellular invasion as compared to the vehicle treated control in LP and HP *Nf1^(−/−)^* MPNST cells. Addition of 200 ng/mL of BMP2 promotes invasion in these cells (**P<0.01), which is blocked by the addition of LDN-193189 in all the tested MPNST cells. Cells were stained with CyQuant/GR dye and the number of invaded cells was quantified by a fluorescence plate reader. **B.** The IC_50_ graph shows the optimal dose of LDN-193189 needed for half maximal inhibition of invasiveness in MPNSTs. **C.** Bar graph of the quantified fluorescence of the cells that invaded through the ECM upon treatment with single agents and in combination. The invasive ability of MPNST cells is greatly reduced by LDN-193189 (0.003 μM), whereas selumetinib only affects cellular invasion at 0.1 μM. The combinatorial treatment results in a statistically significant increase of the inhibitory effect of LDN-193189 on invasion. Based on the BI model, the therapeutic interaction of the combination treatment on invasion was synergistic for both the combinations used. Data presented are mean average of three independent experiments ± S.D. with the corresponding *P*-values (n=3, *P<0.05, **P<0.01, One-way ANOVA followed by Tukey's test for multiple comparisons).

Metastases of MPNSTs occurs in about 40% of patients [23] with the most frequent metastatic sites being lungs, lymph nodes, and liver [38]. BMP2 is over-expressed in many different tumor types [39], notably carcinomas of the prostate, lung, colon, breast, and ovary, where it is known to promote cellular motility, invasion and metastases [40–43]. As expression of *Bmp2* positively correlates with malignancy in neurofibromas [33], increased expression of BMP2 in MPNSTs may promote metastatic characteristics such as cell migration and invasion. Given that BMP2-SMAD1/5/8 pathway is involved in cellular migration and invasion, inhibition of these malignant characteristics by LDN-193189 in MPNST cells was expected. Treatment with LDN-193189 resulted in a statistically significant decrease in the invasive potential of MPNST cell lines (Figure 4A). In addition, recombinant human BMP2 stimulated the invasive activity of the MPNST cells and this effect was abrogated by the addition of LDN-193189, validating that the effects of LDN-193189 on cellular invasion are specific to its inhibition of Type I BMP2 receptors. The IC_50_ of LDN-193189 on cellular invasion is between 0.003-0.005 μM (Figure 4B); concentrations at which LDN-193189 effectively inhibits the activation of BMP2-SMAD1/5/8 signaling based on the western blot analyses (Figure 2A). These results indicate that the anti-invasive effects of LDN-193189 are specific to its competitive inhibition of the BMP2 Type I receptor and the BMP2-SMAD1/5/8 signaling cascade that plays a critical role in the invasiveness of MPNSTs. As MEK1/2 signaling is primarily involved in the regulation of cell viability, growth and proliferation, we did not expect changes in the invasive potential of the MPNST cells upon treatment with selumetinib. However, selumetinib significantly enhanced the anti-invasive effect of LDN-193189 in MPNST cells (Figure 4C). These results indicate that selumetinib, in addition to suppressing the growth of MPNSTs, may also provide a significant benefit by inhibiting the invasiveness of such tumors. Based on the Bliss Independence (BI) model calculations, the combination of LDN-193189 and selumetinib synergistically inhibited MPNST cellular invasion at biochemically relevant concentrations. It is important to note that the combinatorial effects of both agents on the invasive capability of MPNST cells were independent of its effects on cell survival and proliferation, as concentrations of LDN-193189 and selumetinib used in the invasion assay (Figure 4C) were at least 100-fold lower than the concentrations that affect cellular viability (Figure 2B & 2D).

**Figure 5:**
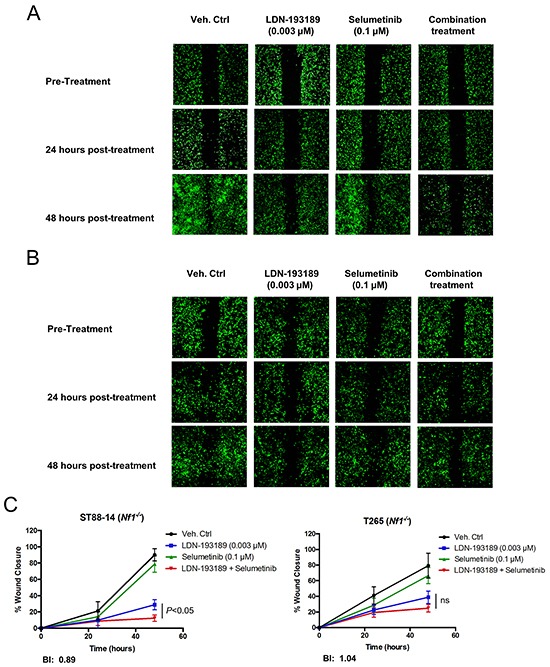
Selumetinib enhances the anti-migratory effects of LDN-193189 in MPNST cells **A.** and **B.** Representative images of the analyzed cellular wound area of low passage ST88-14 (*Nf1^−/−^*) and T265 (*Nf1^−/−^*) cells taken at different time points during the course of the migration assay. Cells were infected with lentiviral GFP to allow for quantification of images. The pre-treatment images were taken upon creation of the wound field and cell migration into the wound field was followed for the next 24-48 hours post-treatment. **C.** The percent quantification of the wound area analyzed using ImageJ was normalized to the pre-treatment wound area for each condition. LDN-193189 (0.003 μM) significantly reduces the motility in both cell lines as compared to the control (*P*<0.0001), however treatment with selumetinib alone does not affect motility. Combinatorial treatment significantly decreases MPNST cellular migration in LP ST88-14 (*Nf1^−/−^*), whereas the combination treatment effect is insignificant in the LP T265 (*Nf1^−/−^*) cells, as compared to treatment with LDN-193189 alone. BI values represent a weakly synergistic to an additive effect of drug combination on motility. Data presented are the average of quantification of the wound areas of at least three independent experiments ± S.D (n=3, Two-way ANOVA for comparing each time point and condition with another, followed by Bonferroni's test for multiple comparisons).

Next, we evaluated the anti-motility effects of combinatorial therapy versus mono-therapy in MPNST cells. As shown in Figure 5, treatment with biochemically effective concentrations of LDN-193189 inhibited migration of both low passage MPNST cell lines. Addition of selumetinib to LDN-193189-treated cells resulted in a statistically significant decrease in the migration of low passage ST88-14 (*Nf1*^−/−^) cells (Figure 5A & 5C). However, the combination treatment did not significantly affect the migratory potential of LP T265 (*Nf1*^−/−^) cells as compared to treatment with LDN-193189 alone (Figure 5B & 5C). The nature of the anti-migratory interaction of LDN-193189 and selumetinib was weakly synergistic to additive in the MPNST cells. This is because the BI model takes into account the independent biological effects of each drug, whereas for statistical analyses combinatorial treatment was compared to treatment with LDN-193189 alone. Similar responses were observed by increasing the dose of LDN-193189 by 3-30 fold in the same cell lines (data not shown).

## DISCUSSION

One of the major questions in development of MPNSTs is the role of signaling pathways other than the Ras-regulated pathways in the transformation and maintenance of these tumors. Given that neurofibromas affect almost all NF1 patients [44, 45] but only 8-13% of these patients develop MPNSTs [46], the molecular events leading from neurofibromas to MPNST formation are unclear. Mutations in *Nf1* and subsequent hyperactivation of downstream signaling pathways of RAS-MEK1/2-ERK1/2 and PI3K-AKT-mTOR are necessary but not sufficient to drive the transformation of neurofibromas to MPNSTs [47, 48]. As carcinogenesis is a multi-step process, we expect changes in multiple signaling pathways and cellular processes to govern the transformation, development and maintenance of MPNSTs. To answer these questions, we used gene expression profiling and identified a druggable target, BMP2-SMAD1/5/8 pathway that was independent of RAS-MEK1/2 regulation in MPNSTs [24]. Our evaluation of expression profiling data of tissue samples from NF1 patients by an independent group [33] showed that BMP2 is significantly up-regulated in both plexiform neurofibromas and MPNSTs with considerably higher levels in MPNSTs [24], suggesting that BMP2 may be one of the key players in the transformation of benign neurofibromas to a malignant phenotype. In this study, analysis of BMP2 mRNA, protein, and secretion showed that BMP2 is expressed in *Nf1*-null MPNST cell lines, independent of passage numbers, and increased expression of BMP2 is dependent on down-regulation of NF1 in MPNST cells. Interestingly, we noticed a much greater increase in the levels of secreted BMP2 upon down-regulation of NF1 (Figure 1F) than the corresponding change in *Bmp2* mRNA expression levels (Figure 1E). These differences suggest that NF1 not only regulates BMP2 transcriptionally, but it may also affect the secretory pathway of BMP2. The absence of NF1 in MPNSTs may lead to increased secretion of BMP2 that promotes malignant characteristics within the tumor microenvironment. Serum levels of BMP2 are used as a prognostic indicator in non-small cell lung cancers (NSCLCs), in which increased serum levels correlate with advanced clinical stage [49]. Based on our results, BMP2 may potentially serve as a prognostic biomarker of the progression to MPNSTs in NF1 patients. Our previous work and this study have demonstrated the significance of targeting the BMP2-SMAD1/5/8 pathway in MPNSTs, which results in reduced malignancy-related properties in MPNST cell lines. By combining the inhibition of BMP2 signaling network with a MEK1/2 inhibitor, we have established the therapeutic utility of combinatorial treatment that results in increased cell death, decreased migration and invasion of MPNST cells. Importantly, this study is an applicable pre-clinical assessment of the proposed combination treatment because all experiments were conducted in very low passage patient-derived MPNST cells that are more relevant to the original tumors' characteristics.

We found increased BMP2 expression and its promotion of malignancy-related characteristics independent of the constitutively active RAS-MEK-ERK pathway in the *Nf1^(−/−)^* MPNST cells [24]. Evaluation of the inhibition efficacy of LDN-193189 and selumetinib by western blot analyses (Figure 2A & 2C) showed that inhibition of BMP2 had no effect on the activation of the MEK-ERK pathway and vice versa. However, small molecular inhibitors of these distinct pathways synergistically interacted to decrease survival, proliferation, invasiveness, and to a limited extent, migration, of MPNST cells. A likely explanation of the synergistic interaction of LDN-193189 and selumetinib in MPNST cells is by cross-talk of BMP2-SMAD1/5/8 and MEK-ERK pathways via another signaling pathway. For example, many studies have described the role of BMP family members including BMP2 in the SMAD1/5/8-mediated activation of the p38 MAPK pathway [50–52]. In fact, Boergermann et al. has shown that inhibition of the SMAD1/5/8 pathway by LDN-193189 results in decreased phosphorylation of p38 MAPK and inhibition of its subsequent targets in murine myoblast cells [53]. Although p38 MAPK and MEK-ERK belong to distinct families of MAPKs, they regulate many of the same downstream effectors such as ATF1, CREB, p65, eIF4E, etc. [54]. It is plausible that the propagated downstream effects of the combinatorial inhibition of MEK1/2 and BMP2 result in targeting of the same substrates involved in cell survival, invasion and migration of MPNST cells.

Throughout these studies, therapeutic efficacy of combinatorial versus single agent treatments was assessed at biochemically relevant concentrations to avoid off-target effects. Thereby, these results not only demonstrate the efficacy of the targeted agents but also highlight the functional roles of the targeted signaling pathways. For example, LDN-193189 inhibits invasion and migration potently at biochemically effective concentrations, whereas its effects on cell growth and survival are possibly off-target effects. The cell growth and survival IC_50_ for LDN-193189 was ~100-fold higher than the biochemically relevant concentration required to inhibit BMP2-SMAD1/5/8 signaling. An independent study of the effects of LDN-193189 on the proliferation of pancreatic cancer cell lines corroborates our results as LDN-193189 decreases the number of viable pancreatic cancer cells [55] within the concentration ranges used in our cell viability experiments. These off-target effects may be mediated by LDN-193189′s effects on receptors or kinases other than BMP Type I receptors. In fact, a study of kinase specificity of LDN-193189 determined that at 1.0 μM concentration (within IC_50_ range in MPNST cells), LDN-193189 affects 24 kinases notably RIPK2, GCK, FGF-R1, etc., independent of its effects on the phosphorylation of SMAD1/5/8 complex [35]. It is important to note that kinases of the MAPK pathway were unaffected by 1.0 μM LDN-193189 in the same study, while results from our western blot analyses concluded that treatment with up to 1.0 μM LDN-193189 does not affect MEK1/2 in MPNST cell lines (Figure 2A, 2B). With the demonstrated lack of adverse side effects of LDN-193189 in animal studies [43], the inhibition of cell growth by LDN-193189 even at higher concentrations may provide an additional therapeutic benefit in treatment of MPNSTs. Additionally, increased cell death in MPNST cells upon combinatorial treatment with LDN-193189 and selumetinib was associated with a dose-dependent increase in PARP cleavage, a marker of apoptosis, as compared to single treatments with LDN-193189 or selumetinib.

We did not expect that selumetinib at biochemically relevant concentrations would affect migration or invasion of MPNST cells, however, results from the *in vitro* invasion studies suggest that selumetinib, in addition to suppressing the growth of MPNSTs, may also provide a significant benefit by inhibiting the metastatic nature of such tumors. The combination treatment with both the candidate agents resulted in a minor yet significant decrease in invasiveness of MPNST cell lines as compared to treatment with LDN-193189 alone. The combinatorial effect of LDN-193189 and selumetinib on the migratory potential of MPNST cells ranges from weakly synergistic to additive. As LDN-193189 is a potent anti-cellular motility agent, it is possible that the effects of the combination therapy with the addition of selumetinib are difficult to interpret in *in vitro* migration assays. Interestingly, low passage *Nf1^(−/−)^* MPNST cells were more sensitive to selumetinib in regards to their migratory potential as compared to the high passage *Nf1^(−/−)^* MPNST cells. We had also found the low passage *Nf1^(−/−)^* MPNST cells to be more sensitive to selumetinib in the cell growth assays (Figure 2D). As low passage cells closely represent the original properties of MPNSTs, increased drug sensitivity of these cells advocates the use of the proposed combination therapy described in this study. Overall, our proposed combinatorial approach of targeting two independent signaling networks with biochemically effective doses of LDN-193189 and selumetinib in physiologically relevant low passage patient-derived MPNST cell lines provides a comprehensive treatment strategy to improve the clinical outcome for MPNST patients.

## MATERIALS AND METHODS

### Cell culture and cell lines

All MPNST cell lines used in this study were maintained in RPMI 1640 medium (Invitrogen, MA) supplemented with 5% fetal bovine serum (Hyclone Laboratories, UT). Low passage human MPNST ST88-14 (*Nf1^−/−^*), and low passage T265 (*Nf1^−/−^*) cells were a generous gift from Dr. Margaret Wallace (Department of Molecular Genetics and Microbiology, University of Florida, FL). Specifically, passages: 8-16 for low passage ST88-14 (*Nf1^−/−^*), and passages: 10-18 for low passage T265 (*Nf1^−/−^*) cell lines were used in this study. High passage human MPNST ST88-14 (*Nf1^−/−^*) cells (from T. Glover, University of Michigan, MI), T265 (*Nf1^−/−^*) cells (from G. De Vries, Hines VA Hospital, Hines, IL), and STS26T (*Nf1^+/−^*) cells (from D. Scoles, Cedars-Sinai Medical Center, CA, USA), were cultured for at least over 100 passages in our lab. STS26T *(Nf1^+/−^*)** cell line was used to establish a stable *Nf1* knockdown cell line using pGIPZ lentiviral *Nf1* shRNA vector, Clone ID: V2LHS_76032 (Open BioSystems, GE Dharmacon, CO). The infected cells were selected with puromycin (1.0 μg/mL) for 8 days post-infection. Selected cells were confirmed by fluorescence microscopy for green fluorescence protein (GFP) expression, and a pooled population was maintained in selection media containing puromycin (0.5 μg/mL) for duration of experiments. Cell lines were periodically checked and found negative for mycoplasma using MycoAlert Mycoplasma Detection Kit (Lonza, Basel, Switzerland). Cultures were propagated for no more than 3 months at a time.

### RNA extraction and quantitative real time PCR

RNA was extracted using the RNeasy Mini Kit (Qiagen, CA). Three batches of total RNA (2.0 μg) for each cell line were reverse transcribed by SuperScript® II First-Strand Synthesis System (Invitrogen). Q-RT-PCR was performed using Power SYBR Green MasterMix (Applied Biosystems) and analyzed on the ABI 5700 Sequence Detection System (Applied Biosystems). The relative fold change was calculated using the CT method as follows: 2^−ΔΔCT^, where, ΔΔCT = (CT *_Bmp2_*- CT *_Gapdh_*) experiment - (CT *_Bmp2_* - CT *_Gapdh_*) control. Statistical significance was determined through student's t-test and a *p*-value of less than 0.05 was considered significant.

### Western blot analyses

Cells grown to 60-80% confluence were washed with cold PBS, scraped and lysed with RIPA buffer supplemented with 1% protease inhibitor cocktail, 1% PMSF (from stock at 10 mg/ml in methanol), 1 mM Na_3_VO_4_, 1 mM Na_4_P_2_O_7_.10H_2_O, and 1 mM NaF. Primary antibodies used were rabbit polyclonal anti-neurofibromin 1:600 (#A300-140A, Bethyl laboratories, TX), rabbit monoclonal anti-phospho-SMAD1/5/8 1:600 (#9516S, Cell Signaling, MA), mouse monoclonal anti-phospho-ERK1/2 1:1000 (#9106S, Cell Signaling), rabbit polyclonal anti-SMAD1/5/8 1:400 (#9106S, Cell Signaling), rabbit monoclonal anti-ERK1/2 1:1000 (#4695S, Cell Signaling) and mouse monoclonal anti-α-tubulin (#T5168, Sigma-Aldrich, MO). Secondary antibodies were conjugated to IRdye infrared dyes (Rockland Immunochemicals, PA). Signal was detected using the Odyssey infrared imaging system and software (Licor Biosciences, NE) and the protein bands were quantified using ImageJ software.

### ELISA test for secreted BMP2 protein

Cell culture supernatants were collected 24 hours post-incubation in RPMI-1640 supplemented with 0.5% FBS. Conditioned media was collected and concentrated using Amicon Ultra-4 Centrifugal filter units with 3 kDa cut-off (Merck Millipore, MA). Secreted BMP2 levels were analyzed using the BMP2 Quantikine ELISA kit (R&D Systems, MN). Duplicates of each sample were analyzed per experiment. A standard curve was generated using optical density (O.D.) of the BMP2 standards provided by the vendor. Secreted BMP2 levels for each sample were calculated against the standard curve and normalized to the standard medium as well as total protein concentration in conditioned media.

### *In vitro* cytotoxicity assays

*In vitro* cytotoxicities of LDN-193189, and Selumetinib, alone or in combination were measured by using 3-[4,5-dimethyl-thiazol-2-yl]-2,5-diphenyltetrazolium-bromide (MTT) (Sigma-Aldrich) in MPNST cell lines. After 48 hours of treatment, MTT was added to a final concentration of 5 mg/mL, and solubilized by addition of 0.1 N HCl in anhydrous isopropanol, after which plates were read within an hour using a microplate reader at 570 nm, with a background subtraction of 690 nm. IC_50_ values were calculated as drug concentrations necessary to inhibit 50% growth compared to vehicle-treated control cells using GraphPad Prism 5 software (GraphPad Software, CA). The anti-tumor interactions between LDN-193189 and Selumetinib were determined by standard isobologram analyses and by evaluating combination index (CI) values as described below.

### Flow cytometry and cell cycle analyses

MPNST cells were treated with specified concentrations of LDN-193189 and/or selumetinib or with vehicle control (DMSO) for indicated time points. Cells were fixed in 70% ethanol overnight. After which, cells were stained with propidium iodide staining solution with RNase A (Cell Signaling Technology) for another 24 hours in the dark at 4°C. Cell cycle analyses and flow cytometry was performed by the Microscopy, Imaging and Cytometry Resources Core at Wayne State University, School of Medicine, using BD LSRII.

### Migration and invasion studies

The effects of LDN-193189 and selumetinib alone, or in combination on MPNST cell invasion were determined using the CytoSelect™ 96-well cell invasion assay kit (Cell Biolabs Inc.) as per manufacturer's instructions. Cells were pretreated overnight with indicated concentrations of LDN-193189, selumetinib, alone or in combination before seeding to the chambers. FBS used at 10% as the chemoattractant (150 μl), was added to the feeder tray. After 18 h of incubation, lysate was transferred to a black walled plate with optical bottoms (Sigma-Aldrich) and fluorescence measurements were performed in a fluorescence plate reader Spectramax I3X (Molecular Devices, CA) at 480 nm/520 nm. The effects of LDN-193189 and selumetinib on motility was investigated by using CytoSelect™ Wound Healing Assay Kit as per manufacturer's instructions (Cell Biolabs, Inc., CA). Cells were seeded at 4-5×10^4^ cells/well in 24-well plates containing inserts aligned in the same direction. Migration of cells into the wound field was followed for the next 24-48 hours after removal of the inserts. In order to visualize the cell migration images by fluorescence, low and high passage T265 (*Nf1^−/−^*) and ST88-14 (*Nf1^−/−^*) cells were infected with the GFP expressing lentiviral plasmid. Cell migration activity was determined by quantification of the wound field area by ImageJ Software (Media Cybernetics, MD) using fluorescent images, photographed by Olympus IX71 fluorescent microscope. Wound healing activity is expressed as the percentage filling the wound field from three independent experiments.

### Statistical analyses and synergy calculations

Paired t-test or ANOVA was used to determine the significant differences at 95% confidence interval. One and two-way ANOVA were followed by post-hoc tests, as indicated. Drug/drug synergy was evaluated by the Chou combination index (CI) using Compusyn software (ComboSyn Inc., NJ), where CI < 1, CI = 1, and CI > 1 indicate synergistic, additive, and antagonistic effects, respectively. Bliss Independence (BI) model was used to calculate the therapeutic interactions of the combination of candidate agents on migration and invasion because it assumes different, independent, mutually nonexclusive sites of action for the candidate agents [56, 57], which is the case in this study. BI model is applied as follows: BI = ((F_a_ + F_b_) - (F_a_ × F_b_))/F_ab_ where: F_a_ = fraction of effect of drug A, F_b_ = fraction of effect of drug B, (F_a_ + F_b_) - (F_a_ × F_b_) = predicted sum of the effects of combination treatment, and F_ab_ = actual effect of combination therapy found experimentally.
